# Current Progress and Challenges of Using Artificial Intelligence in Clinical Dentistry—A Narrative Review

**DOI:** 10.3390/jcm12237378

**Published:** 2023-11-28

**Authors:** Zinovia Surlari, Dana Gabriela Budală, Costin Iulian Lupu, Carmen Gabriela Stelea, Oana Maria Butnaru, Ionut Luchian

**Affiliations:** 1 Department of Fixed Protheses, Faculty of Dental Medicine, “Grigore T. Popa” University of Medicine and Pharmacy, 700115 Iasi, Romania; zinovia.surlari@umfiasi.ro; 2 Department of Implantology, Removable Prostheses, Dental Prostheses Technology, Faculty of Dental Medicine, “Grigore T. Popa” University of Medicine and Pharmacy, 700115 Iasi, Romania; dana-gabriela.bosinceanu@umfiasi.ro; 3 Department of Dental Management, Faculty of Dental Medicine, “Grigore T. Popa” University of Medicine and Pharmacy, 700115 Iasi, Romania; 4 Department of Oral Surgery, Faculty of Dental Medicine, “Grigore T. Popa” University of Medicine and Pharmacy, 700115 Iasi, Romania; 5 Department of Biophysics, Faculty of Dental Medicine, “Grigore T. Popa” University of Medicine and Pharmacy, 700115 Iasi, Romania; oana.maria.butnaru@umfiasi.ro; 6 Department of Periodontology, Faculty of Dental Medicine, “Grigore T. Popa” University of Medicine and Pharmacy, 16 Universității Street, 700115 Iasi, Romania; ionut.luchian@umfiasi.ro

**Keywords:** artificial intelligence (AI), machine learning, neural networks (NNs), digital dentistry

## Abstract

The concept of machines learning and acting like humans is what is meant by the phrase “artificial intelligence” (AI). Several branches of dentistry are increasingly relying on artificial intelligence (AI) tools. The literature usually focuses on AI models. These AI models have been used to detect and diagnose a wide range of conditions, including, but not limited to, dental caries, vertical root fractures, apical lesions, diseases of the salivary glands, maxillary sinusitis, maxillofacial cysts, cervical lymph node metastasis, osteoporosis, cancerous lesions, alveolar bone loss, the need for orthodontic extractions or treatments, cephalometric analysis, age and gender determination, and more. The primary contemporary applications of AI in the dental field are in undergraduate teaching and research. Before these methods can be used in everyday dentistry, however, the underlying technology and user interfaces need to be refined.

## 1. History

Since the dawn of scientific inquiry, scientists and engineers have labored to unravel the mysteries of the human brain, a tangled web of billions of neurons relaying information throughout the body [1].

The human brain can be conceptualized as a complex network of interconnected neurons that communicate with one another, transmitting electrical impulses throughout the entire body [2]. The scientific community continues to face challenges in the development of a model that accurately replicates the complexities of the human brain. For several years, diligent researchers have been devotedly engaged in the development of a concept known as “Artificial Intelligence” [3].

The concept of “Artificial Intelligence” (AI) dates back to the 1950s; it describes the creation of robots that can mimic human intelligence and behavior [4].

The direct and indirect expenses of treating oral and dental diseases, which are among the most common health problems in the world, totaled more than USD 500 billion in 2015 [5]. This burden of oral and dental diseases is expected to increase due to demographic and epidemiological dynamics, but there is a relatively small number of people who are trained to provide oral and dental care. This is putting additional strain on already overburdened health care systems and endangering the accessibility and affordability of oral and dental health services.

AI and its applications have been seen as both a blessing and a curse throughout the past seven decades. During this time, there have been multiple instances in which technological advancements have fallen short of expectations. However, the past ten years represent the golden age of artificial intelligence. The current state-of-the-art artificial intelligence-based natural language modeling has become so convincing that readers cannot tell the difference between human- and machine-written text. The second innovation is facial-recognition systems. The impact of AI-based technology on many facets of society, including healthcare and politics, has reached a tipping point. One of these fields is dentistry [6,7]. AI’s potential for increased effectiveness, safety, and efficiency in oral and dental care is particularly promising because it will enable better care to be provided to more people in less time [8].

**Figure 1:**
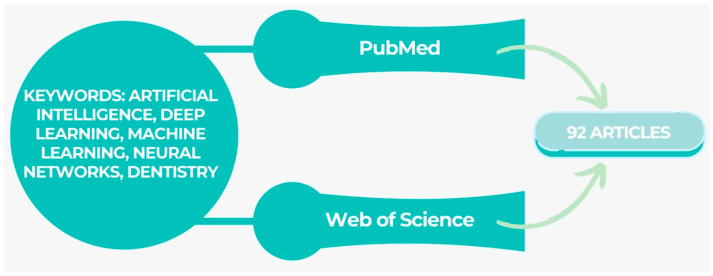
Article selection for the study.

This article aimed at reporting on the application and performances of AI models that have been designed for application in dentistry. Prominent internet databases, including PubMed and Web of Science, were utilized to retrieve publications pertinent to the research inquiry that were published between January 2000 and 2023. The majority of the experiments incorporated in the analysis were conducted utilizing convolutional neural networks. Over the past five years, there has been a notable surge in the quantity of scholarly articles documenting the utilization of artificial intelligence (AI) models, as can be seen in Figure 1 below.

## 2. Types of AI

In 1978, Richard Bellman, a scholar in applied mathematics, provided a definition for artificial intelligence (AI) as the mechanization of cognitive processes inherent to human thinking skills [9]. These processes comprise the acquisition of knowledge, the formulation of choices, and the resolution of difficulties.

**Figure 2:**
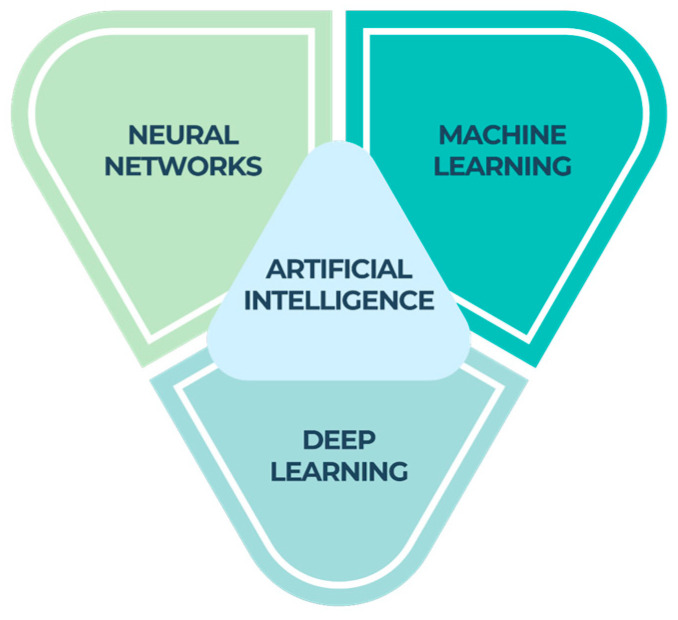
Fundamental aspects of artificial intelligence.

In contemporary society, the term “artificial intelligence” pertains to any machinery or technological system that possesses the capability to replicate human cognitive abilities, such as problem solving. In order to derive a comprehensive understanding of artificial intelligence (AI), it is important to familiarize oneself with certain fundamental features, which are illustrated in Figure 2.

Artificial intelligence (AI) is defined as the capacity of machines to demonstrate a distinct sort of intelligence. The objective of this study was to design and construct machines capable of acquiring knowledge from data in order to effectively address various problems [10]. Clinical outcomes have not been shown to improve using current research and performance metrics for evaluating machine learning (ML) models. While models may be able to precisely identify disorders in images or generate assumptions based on electronic health records, it is the practitioner’s suggestions for therapy that ultimately determine the impact on clinical outcomes by taking into account the patient as a whole, the net benefits of available treatment options, and the patient’s willingness to comply with treatment [10].

⇒ Machine learning (ML), a component of artificial intelligence (AI), relies on algorithms to make predictions by analyzing a given dataset. The objective of machine learning is to enable machines to acquire knowledge from data in order to autonomously address problems without the need for human intervention [11].
⇒ Neural networks (NNs) are widely recognized as a prominent category of machine learning (ML) models. They have demonstrated superior performance compared with traditional ML techniques, particularly when dealing with intricate data formats such as photography or language. Neural networks refer to a collection of techniques that perform signal computation through the use of artificial neurons. The primary objective of neural networks is to develop computational models that simulate the everyday activities of the human brain [12].

NNs can transfer any input (for example, an X-ray of a decaying tooth) to a given output (such as “decayed tooth”) given a set of mathematical restrictions. With sufficient data and processing power, these NNs can be educated to precisely reflect the underlying statistical structures inherent in the input. When being trained, an NN is repeatedly fed data points along with their labels (for a classification task) or numerical outputs (for a regression task) [13]. The weights of the model, which represent the interconnections between the neurons, are thus optimized in an iterative process to reduce the prediction error. By feeding a new data point through a trained NN, it is possible to predict the result of previously unseen data.

⇒ Deep learning is a fundamental element of machine learning that uses a multi-layered cognitive matrix within a deep neural network to examine and interpret incoming data. The objective of deep learning is to develop a neural network that autonomously recognizes patterns in order to enhance the process of feature detection [14].

Even if a model is very good at predicting periodontal disease, the success of the clinical outcome depends heavily on the practitioner’s ability to change, for example, a patient’s smoking behavior. Therefore, it is suggested that an evaluation approach be devised that takes into account occurrences subsequent to the ML model’s outputs in order to provide a fuller picture of the impact the model has on patient outcomes.

In order to keep up with changes in the local area in which they are working and ensure optimal performance and patient safety, ML systems require regular monitoring, retraining, and maintenance.

Data-driven machine learning models are often praised as objective decision-making systems that are devoid of human biases. Nevertheless, inadvertent integration of human biases into these systems might occur, posing a particular challenge in the case of black box algorithms where identification and correction of such biases can be particularly challenging. There has been a growing recognition of the potential for ML models to perpetuate or amplify pre-existing social prejudices and health inequities.

The capacity to ultimately enhance patient care and provide accurate diagnoses has reshaped the healthcare industry. The ability to coach patients on how to change their behavior while still carrying out dental procedures is in high demand. This is especially true in the field of pediatric dentistry.

## 3. Applications in Dentistry

Artificial intelligence is playing a pivotal role in the modernization of conventional practices within the field of dentistry. Artificial intelligence technologies are frequently employed in the development of automated software programs that enhance the efficiency of diagnostics and data administration within the field of dentistry. Primarily, clinical decision support systems serve as tools that aid and direct professionals in making improved decisions [15]. These methods have been utilized to enhance the accuracy of diagnoses, aid in the development of treatment plans, and facilitate the predictions of prognoses. The increasing demand for these systems can be attributed to their efficacy in delivering explanations and logical reasoning.

Many AI-based tools are currently employed to streamline and automate previously labor-intensive dental procedures. These technologies provide a number of helpful services, including enhanced diagnosis accuracy, disease prediction, and treatment recommendations, to simplify the dentist’s workload. Artificial intelligence is used in many different areas of dentistry, from cavity detection to gender determination in forensic dentistry [16].

The use of AI has completely changed the dental sector and simplified dentists’ jobs. The primary function of AI-powered clinical decision support systems is to provide assistance to doctors and nurses. Any computer program that deals with medical data or the medical knowledge necessary to interpret such data and is designed to aid health professionals in making clinical decisions falls under the umbrella term of a “clinical decision support system” [17].

The utilization and impact of artificial intelligence (AI) has experienced a notable surge across various industries, encompassing the field of dentistry. The ability to replicate human intellect in order to do intricate forecasts and decision making within the healthcare industry is evident. Convolutional neural networks (CNNs) and artificial neural networks (ANNs) have demonstrated diverse uses within the field of dentistry. Potential future uses of this technology were examined in the context of scheduling, patient care, drug–drug interactions, prognostic diagnosis, and robotic surgery.

Nevertheless, before integrating AI models into regular clinical practices, it is crucial to conduct additional assessments to ascertain the cost-effectiveness, reliability, and suitability of these models.

Artificial intelligence possesses the potential to revolutionize the medical and dentistry professions by offering solutions to various clinical problems, hence facilitating the work of physicians The integration of AI inside the dental business is not yet commonplace.

### 3.1. AI in Diagnostics and Radiology

In intraoral periapical films, Chen et al. [18] used CNNs to count teeth and later identify them. The model’s accuracy was remarkable. The calculation of precision and recall on a test dataset involves determining the intersection-over-union (IOU) value between the identified and actual elements. The findings indicate that the precision and recall metrics both surpass 90%, while the average intersection-over-union (IOU) value between identified units and ground facts likewise attains 91%. It became clear from the study’s findings that AI tools streamlined the clinical workflow. They can skip entering the information by hand. Dentists can save time and effort with these automated systems because they enable dental charts to be entered digitally.

In their study, Lee et al. [19] reported the use of CNN algorithms for detecting and diagnosing dental caries on periapical radiographs, demonstrating the efficacy of AI technology in this area. The assessment precision of models representing premolars, molars, and both premolars and molars were found to be 89.0% (with a confidence interval of 80.4–93.3), 88.0% (with a confidence interval of 79.2–93.1), and 82.0% (with a confidence interval of 75.5–87.1), respectively. The application’s results were noticeably impressive. Studies using deep learning models for the detection and localization of dental lesions in near-infrared transillumination (NILT) pictures have shown encouraging results, as was the case in research by Casalegno et al. [20]. The study successfully obtained an average intersection-over-union (IOU) score of 72.7% for a 5-class segmentation task using a limited dataset of 185 training samples. Furthermore, the specific IOU scores for proximal and occlusal carious lesions were found to be 49.5% and 49.0%, respectively.

Deep learning techniques have been employed by Talpur et al. [21] in a study for the analysis of dental pictures for the purpose of diagnosing dental caries, encompassing three distinct types: proximal, occlusal, and root caries. The Neural Network Backpropagation algorithm, one of the deep learning algorithms, gives a maximum accuracy of 99%. A study by Hung et al. [22] on the use of AI to predict root caries showed promising results. Among the various machine learning algorithms that have been developed, it was seen that the support vector machine algorithm exhibited the highest level of performance. This was evidenced by an accuracy rate of 97.1%, a precision rate of 95.1%, a sensitivity rate of 99.6%, and a specificity rate of 94.3% in effectively recognizing cases of root caries.

Using NILT pictures, Schwendicke et al. [23] demonstrated strong performance of these AI-based models for the diagnosis of dental caries. The prevalence of caries lesions at the tooth level was found to be 41%. The sensitivity and specificity values were reported as 0.59 (95% CI: 0.47–0.70) and 0.76 (95% CI: 0.68–0.84), respectively. Upon visual examination of the model’s predictions, it was shown that the model exhibited sensitivity towards regions that were impacted by caries lesions. The results of the study indicate that the moderately deep CNNs had a satisfactory selective capacity in detecting caries lesions.

The use of CNNs for a single or extra root on Cone Beam Computed Tomography (CBCT) images and panoramic radiographs of 760 mandibular first molars from 400 patients was reported by Hiraiwa et al. [24]. The study involved the segmentation of image patches of roots from panoramic radiographs, which were then utilized in a deep learning system. The main goal was to evaluate the diagnostic capabilities of this system in classifying root morphology. Additional roots were detected in 21.4% of distal roots based on the analysis of CBCT images. The deep learning system exhibited a diagnostic accuracy rate of 86.9% in discerning the presence of additional roots in distal roots, as opposed to a single root.

When used with panoramic dental radiographs, CNNs developed by Ekert et al. [25] were successful in detecting apical lesions (ALs). The positive predictive value (PPV) obtained was 0.49 (standard deviation = 0.10), whereas the negative predictive value (NPV) achieved was 0.93 (standard deviation = 0.03). The study found that the level of sensitivity in molars was significantly greater compared with other types of teeth, while the level of specificity was comparatively lower.

The deep learning technique was also used by Murata et al. [26] to analyze panoramic radiographs for signs of maxillary sinusitis. This system’s diagnostic performance was satisfactory. The deep learning system exhibited a high level of diagnostic performance in the detection of maxillary sinusitis on panoramic radiographs, with an accuracy rate of 87.5%. Additionally, the system demonstrated a sensitivity of 86.7% and a specificity of 88.3%. When compared with the performance of seasoned radiologists, these findings were consistent with the study of Kim et al. [27].

The ability of artificial intelligence to detect and diagnose osteoporosis was assessed by Lee et al. [28]. Using panoramic radiographs, researchers tested a CAD system based on a deep CNN and found it to be highly effective at detecting osteoporosis. When compared with the detection rates achieved by experienced oral and maxillofacial radiologists, the performance of the CAD system was significantly higher. Using deep CNN, Lee et al. [29] found comparable success in diagnosing osteoporosis in dental panoramic X-rays.

Table 1 summarizes some research using AI-based models for diagnosis in several areas of dentistry, as found in the literature research.

### 3.2. AI in Endodontics

Endodontists rely heavily on the interpretation of diagnostic imaging, including intraoral radiographs, cone beam computed tomography scans, and orthopantomography images, for both treatment planning and diagnosis. CNNs with multiple layers may prove useful in the analysis of X-ray images using AI, as this method is based on simultaneously verifying adaptive image features and performing image classification, eliminating the need to input predefined image signs for calibration of the identification process [30]. Root canal therapy can be performed without resorting to surgery if dentists are familiar with root morphologies and root canal anatomy. In addition to identifying mistakes made when mapping out new canals, AI can recognize morphological anomalies.

Three-dimensional tooth segmentation was automated using the CNN method by Lahoud et al. [31]. The scientists analyzed 433 CBCT radiographic segmentations of teeth to create a swift, efficient, and reliable clinical benchmark; they found that AI performed as well as a human operator but at a far faster rate.

The lesion diagnostic effectiveness and dice coefficient indices of a multilabel segmentation were evaluated between a morphologically constrained Dense U-Net and established clinical image analysis methods by Zheng et al. [32]. The researchers discovered that the unique deep learning technique improved CBCT segmentation and the accuracy of pathological detection, despite the limited sample size.

Extremely seldom do teeth that have received endodontic treatment experience “vertical root fractures (VRFs)”. Radiographic VRF determination is challenging and may require more cutting-edge tools. Fukuda et al. [33] suggested that CNNs are a promising method for detecting and quantifying VRFs on panoramic radiographs. Kositbowornchai et al. [34] used a probabilistic neural network design to predict with 95.7% accuracy whether a tooth root was healthy or had a vertical root fracture.

When performing root canal therapy, it is imperative that an appropriate working length (WL) be chosen. Inadequate WL determination commonly leads to instrumentation beyond the apical foramen, flare-ups, periapical foreign body reactions, and poor microbiological control [35]. Radiography, digital tactile sense, and patient responses to a file or paper point are all viable options for locating the apical foramen and determining the WL [36]. The use of digital technology has demonstrated both advantages and disadvantages in locating the apical foramen. In light of these findings, it may be prudent to recommend the use of AI for working length verification and apical foramen detection by non-experienced dentists or dentists without endodontic specialization during root canal therapy.

According to a recent systematic review, the accuracy of AI for detecting vertical root fractures ranges from 73.6% to 96.6%, and the accuracy of AI applied over CBCT data is even higher in the case of untreated root canals [37]. However, when it comes to detecting root fractures in instances of obturated root canals, AI applications perform significantly better than traditional radiographs [37]. A recall of 0.75, precision of 0.93, and F-measure of 0.83 were used to characterize the performance of the AI root fracture detector on the panoramic images [33].

Using deep learning segmentation for identifying periapical lesions, AI achieved a detection accuracy of 0.93 in a study by Setzer et al. [38]. The accuracy of an automated periapical lesion classification system using convolutional neural networks was 70% [39]. However, the accuracy was higher for datasets of images containing large lesions and without such lesions than for datasets containing small lesions and without such lesions.

Analysis of panoramic pictures using a convolutional neural network has been shown to be highly accurate and sensitive (84.37 ± 2.79 and 81.26 ± 4.79, respectively) for detecting a damaged endodontic file within the root canal [40].

Another potentially game-changing application of AI in endodontic regeneration therapies is the capacity to assess stem cell viability and survival [28]. Pulp stem cells cultivated in human platelet lysate exhibited higher viability than those cultured in fetal bovine serum or human platelet-rich plasma, according to analysis performed using a hybrid machine learning method [41]. Endodontists’ current line of thinking on the subject centers on creating artificial intelligence-guided protocols for regenerating tooth pulp stem cells for possible future therapeutic application [42].

Currently, AI models should be seen as supplementary tools for the sake of a potentially improved decision-making process in endodontic practice. The training of future endodontic professionals may benefit from using AI features.

### 3.3. AI in Periodontology

Attempts to use AI in the study of periodontal diseases have also been made. Researchers in the fields of periodontology and mucosal disorders have examined the makeup of saliva and oral microbes. In recent research, NNs have been proven to be useful for distinguishing yeasts from other salivary bacteria [43]. Rather than identifying the specific species responsible for the production of inflammatory oral compounds, this method uses the concentration of methyl mercaptan in the oral air as an indicator of oral odor, in addition to the peak areas of restriction fragment length polymorphisms (T-RF) of the 16S rRNA gene, as data for supervised machine learning methods. Methyl mercaptan, a volatile sulfur-containing molecule that causes yeast infections in the oral cavity, has been modeled using T-RF frequencies and proportions for classification [44].

Using periapical radiographs, Lee et al. [29] constructed a deep learning algorithm to categorize periodontally damaged posterior teeth. They demonstrated a diagnosis accuracy of 81% for premolar teeth with periodontal disease and 76.7% for molar teeth with periodontal disease.

High sensitivity, specificity, and accuracy were achieved for the various stages—all greater than 0.8 [45]; another study showed a diagnostic accuracy of 0.85, with no significant differences in the radiographic bone loss (RBL) percentage measures determined by the deep learning and examiners.

Other models have employed DL to identify RBL, or to calculate the RBL% from the panoramic radiographs, and to assign periodontitis staging from panoramic radiographs [46,47,48]. Although these models using panoramic radiographs have demonstrated acceptable accuracy and repeatability in determining bone level, panoramic radiographs are not recommended due to their distortion, overlap, and low resolution [49,50].

### 3.4. AI in Prosthodontics

The durability of dental restorations is finite. The longevity of dental materials and many dental traits are determining factors. In a recent study conducted by Aliaga et al. [51], artificial intelligence (AI) analysis was performed on a collection of charts, notes, and imagistic information. The objective of the study was to identify the optimal material for cavity repair and to assess its long-term monitoring capabilities during the reconstruction process.

An early study [52] published in prosthodontics by Chen et al. also developed a clinical decision support system model for the precise design of removable partial dentures. Patients’ oral disorders and denture components are represented in an ontological paradigm, and the degree of resemblance between an input patient and a standard ontology example is determined using an inverse similarity method.

One example of a novel digital process is the use of intraoral scanners in implant prosthodontics to record the position of implants using a scan body, essentially a digital replacement for the traditional implant transfer [53,54,55].

The precision of modern intraoral scanners is such that the traditional imprint can be dispensed with in favor of using trays and materials, which is better for the patient and the overall prosthetic workflow [53,56]. Taking the impression is more convenient for the practitioner, causes less pain for the patient, can be performed in less time, and yields consistent outcomes [56,57]. The time and money spent communicating with the lab is minimized, and the processes are made easier. The introduction of digital technologies entails the need to embrace new standards; however, this is not always easy for the dentist and dental technician, especially if they are ‘native analogue’.

Automatic set-up designers for complete dentures, automatic framework designs for removable partial dentures, and determining the emergence profile in implantology are just a few examples of how combining AI technologies in prosthodontics could lead to a wide variety of novel options [58]. Finally, as an instructional tool, AI offers the chance to aid less experienced undergraduate students in their professional development [59].

In theory, there is nothing limiting us from employing AI’s power in prosthodontics. However, interesting dental applications are sometimes prevented due to the perception that the market is too small for the dental business by the same economic concerns that drive the development and widespread adoption of new AI technology in the most economically viable fields. However, the fact that AI science is making strides forward is encouraging.

### 3.5. AI in Orthodontics

Multiple research investigations have shown that ML has the ability to aid in orthodontic diagnosis, treatment planning, and decision making at a high level of quality. When compared with conventional methods, AI facilitates faster, more accurate, and more objective results.

In contrast to more conventional ML models, CNN-based models have proven to be a useful tool in real-world clinical settings. The CNN model was initially used by Japanese researchers to locate 10 landmarks among 153 lateral cephalograms. However, the small sample size and inherent measurement bias compromised the accuracy [60].

With the use of intra-rater and inter-rater accuracy in calibration, Kunz et al. increased the dataset size to 1792 distinct cephalograms for training purposes [61]. Other than the incisor inclination, the CNN model’s predictions for the 11 angles and distances utilizing these coordinates showed no statistically significant changes.

Bone age prediction using cervical vertebrae maturity is used to determine the degree of divergence a patient exhibits from normal growth [62]. Vertebral bodies with trapezoidal tapers and modest edge concavities are used in age prediction methods [63]. The sensitivity, specificity, and accuracy of ML-based approaches for diagnosing vertical and sagittal skeletal maturation all exceed 90% [64].

The debate between orthodontic procedures involving extractions and those that do not have yet to be resolved. Dental protrusion, crowding, and jaw dysplasia are currently treated with orthodontic extraction [65].

For deciding whether or not to perform an extraction, Xie et al. proposed decision-making models based on ANNs [66]. Using backpropagation, ANN achieved a perfect score on the training set, but it could only achieve 80% on the testing set.

Artificial intelligence-based algorithms have indicated that inadequate lips and lower incisor inclination are the key determinants of whether teeth should be extracted before orthodontic treatment [66]. Artificial intelligence models, however, have only been utilized to determine whether extractions are necessary based on cephalometric results and other data.

The use of AI in dentistry is rapidly developing, which bodes well for the future of accurate disease diagnosis, treatment planning, and administration. Moreover, AI should be seen as an aid to diagnosis and therapy by physicians, not a danger. More clinical trials including the use of AI in orthodontics are needed to transform the current state of orthodontic care.

## 4. Emerging Trends and Patterns

Artificial intelligence (AI) holds great potential in the fields of medicine and dentistry. Gaining a more thorough and accurate understanding of our patients’ health and problems would facilitate the more precise allocation of therapies based on predictions. More accurate and individualized care with improved efficacy and safety is possible. In addition, AI has the potential to enable the delivery of services at greater scale and efficiency, using a more diverse workforce; this would go a long way towards relieving worker shortages around the world and broadening access [67].

This analysis found that the majority of contemporary applications for artificial intelligence (AI) in dentistry are restricted to prototypes of automated diagnostics, particularly in dental imaging and radiology [68] and in classification tools, such as those for periodontally damaged teeth or caries.

More and more disciplines of dentistry, such as prosthetics research, are making use of AI for more effective data processing. Due to the ease with which digitally coded images could be imported into AI systems [69], dental image categorization and data processing from area scanning techniques were the earliest uses of AI in dentistry. Diagnostic applications of AI are still being refined. Future advances in patient-centered individualized treatment [68] can be facilitated by the use of AI technologies in dentistry, which have the potential to become key to the triangle of patient data management, health care applications, and services. In Table 2 below we tried to summarize important research regarding AI applications in all fields of dentistry.

The merging of AI with precision medicine has the potential to fundamentally transform the medical industry. Artificial intelligence augments the innate intelligence of clinicians by using complex computing and inference to provide insights, allowing the system to think and acquire knowledge, and to enable doctors to make choices. Personalized diagnoses and prognoses are made possible by combining information from patients’ symptoms, clinical history, and lifestyle with information from their DNA. The recent literature suggests that translational research exploring this convergence will help solve the most difficult challenges facing precision medicine [81].

Changes and advancements in the development and use of AI in healthcare are being fueled by the digitization of health-related data and the fast adoption of technology. The use of AI in healthcare may be hindered, however, by issues like multidimensional integration of information, safety, federated learning (which necessitates essential developments in fields like privacy, large-scale artificial intelligence, and distributed optimization), model performance, and bias [82].

Additionally, it would be interesting to match artificial intelligence both with smartphone applications [83] or computer software [84] in order to test more easily the reliability of AI-based programs in daily clinical practice.

Recent studies have shown that AI has a lot of potential in the medical field. It is probable that AI will continue to play an increasingly important role in health, both at the individual and population levels, and efforts are underway to overcome obstacles in this area [85].

Previous research has linked AI to areas of dentistry [86] other than prosthodontics. Root fractures, periapical diseases, and root morphology can all be identified using radiologically driven AI evaluations that aid in tooth preservation [87,88]. Clinical and radiographic periodontal variables are predicted through AI technology, enabling the easier evaluation of disease progression in periodontology. Radiological images can be analyzed by AI in oral surgery to look for pathological alterations such cysts and bone tumors [89]. As an added bonus, AI may find use in the field of implantology. AI-based treatment planning in computer-aided design (CAD)/computer-aided manufacturing (CAM) implant dentistry may be of tremendous use in streamlining virtual 3D treatment planning and, eventually, in the robotic insertion of dental implants [89].

In this age of widespread epidemics, many youngsters are affected by oral health issues. The key to a child’s best health is early diagnosis, prevention, and treatment of these diseases. The development of artificial intelligence (AI) has accelerated greatly in recent years. Thus, we see AI’s penetration even into domains hitherto deemed to be best left to human professionals. The demand for dental professionals who possess the ability to effectively administer treatment procedures while also offering appropriate counseling on patient behavior is particularly high in the field of pediatric dentistry [90].

We can now declare categorically that AI will never be able to fully replace dental clinicians or pediatric dentists, but it will be a valuable asset in all facets of dental health, from prevention to restoration to diagnostics.

Artificial intelligence has been widely employed in pediatric dentistry to help less experienced dentists make more precise diagnoses. These models are useful at both the individual and societal levels, as they may be used to classify children into risk groups, identify and number teeth, diagnose early ectopic eruption, determine a child’s age, and much more [91].

AI can be utilized as a supplemental tool, preserving the human element and reaffirming the authority of dentists and pediatric dentists over treatment protocols and judgements. However, the day when dental practitioners and AI might combine for better patient care is not far distant.

It is important to remember that people have previously placed high hopes in AI technologies and that the history of AI is marked by both enthusiasm and disappointment. The current state of AI in the medical and dental fields has not lived up to all expectations. When AI is trained on insufficient data, it can become biased, and its resulting application can only be used in specific contexts. Accuracy metrics have been the primary focus of medical and dental AI research rather than establishing actual clinical utility for patients, providers, or the healthcare system as a whole.

Advancements in artificial intelligence have exploded in the last decade. However, how AI research can aid in the prevention, detection, and treatment of dental illness remains to be seen.

Additionally, the usage of AI in dentistry still lags behind that in medicine. Although AI applications in dentistry have yet to fully realize their full potential, they show great promise as a potential fundamental resource for gathering, analyzing, and planning patient-related datasets in order to provide patient-focused and specific treatment.

Although the results of the evaluated research are promising, this particular study does have certain limitations. The literature’s quality assessment acknowledged the potential presence of bias. It is important to take into account the intricacy of a specific system or mechanism, the associated expenses, and the necessary equipment for each configuration, as well as the training prerequisites for each artificial intelligence model. Additional investigation, exploration, and application is necessary. The desired results have not yet been attained due to the lack of precise and adequate data. In summary, obstacles are present in both technological and ethical dimensions.

## 5. Conclusions and Perspectives

The future of dentistry is bright, and the introduction of AI-powered tools is eagerly anticipated. AI has the ability to disrupt and reinvent procedures across the board in dentistry; however, the adoption of AI technology in prosthodontics is currently slow. When it comes to performing repetitive tasks and processing massive amounts of data for classification purposes, AI systems dominate. It is expected that AI algorithms will aid in the study of specific patient cases and provide support in evidence-based dental decision making, especially for less experienced practitioners. It may be possible to provide more uniform treatment methods that leave room for customization.

Although this has a lot of potential, we cannot discount the challenges faced by AI systems. Based on the results of this comprehensive literature review, it is clear that the investigations should be regarded as preliminary and experimental and that the procedures employed in these research projects are not yet ready for regular clinical usage in the field of prosthodontics.

The primary applications of AI in the dental field are currently in undergraduate teaching and research. Before these methods can be used in everyday dentistry, however, the underlying technology and user interfaces need to be refined. There are still several necessary intermediate steps that need to be achieved before AI may be widely adopted. Future research on AI technology in reconstructive dentistry should rigorously investigate not only the technical potential but also the financial implications and moral or ethical issues.

## Figures and Tables

**Table 1:** Main recent publications summarizing the outcomes of AI.

Authors	Goals of Research	Outcomes
Chen et al. [18]	Artificial intelligence-based algorithm for detecting proximal dental caries	The accuracy of proximal caries diagnoses may be raised with the help of this neural network.
Lee et al. [19]	Artificial intelligence deep learning technique for finding caries	Dental caries identification on periapical radiographs was significantly improved with the use of this DCNN (deep convolutional neural network)-based system algorithm.
Casalegno et al. [20]	Dental lesion detection and localization in near-infrared transillumination (TI) pictures using an artificial intelligence-based algorithm	This CNN-based algorithm performed impressively in terms of speed and accuracy in detecting caries.
Talpur et al. [21]	Proximal caries diagnosis using an artificial intelligence model	Proximal caries diagnosis may benefit from the use of this neural network.
Hung et al. [22]	Root caries analysis with AI	The results of this model are satisfactory enough to permit its use in clinical settings.
Schwendicke et al. [23]	Caries lesion detection in near-infrared light transillumination (NILT) pictures using convolutional neural networks (CNNs)	The model’s selective power to identify caries lesions was found to be acceptable.
Hiraiwa et al. [24]	Artificial intelligence for the identification of first molar root shapes in the mandible	The deep learning system performed exceptionally well in determining whether or not the distal roots of mandibular first molars included a single or an additional root.
Ekert et al. [25]	An artificial intelligence system based on CNNs for the diagnosis of apical lesions (ALs)	The AI system based on a deep CNN was able to identify apical lesions.
Murata et al. [26]	Diagnostic artificial intelligence for maxillary sinusitis	Diagnostic accuracy was improved through the AI-based deep learning method.
Kim et al. [27]	Maxillary sinusitis diagnosis using CNNs	In both datasets, the average area under the curve achieved by the AI-based (CNNs) was statistically superior to that achieved by the radiologist.
Lee et al. [28]	Classification of osteoporosis characteristics using deep neural networks	This DCNN approach could be useful and reliable for automating the screening of patients for osteoporosis.
Lee et al. [29]	The objective of this study was to assess the discriminatory capabilities of deep convolutional neural networks (CNNs) when used with different transfer learning techniques. The focus is on classifying distinct characteristics of osteoporosis in digital panoramic radiographs (DPRs).	The findings indicate that the utilization of suitable deep convolutional neural network (CNN) structures in conjunction with transfer learning methods has successfully addressed the challenge posed by a limited training dataset of images. Moreover, the results show that dual-energy X-ray absorptiometry (DXA)-based projection radiography (DPR) images have promise for the pre-screening of osteoporosis.

**Table 2:** Main recent publications in all dentistry fields.

Authors	Area of Dentistry	Year of Publication
Hung K et al. [70]	Oral diagnosis	2022
Ezhov et al. [71]	Oral diagnosis	2021
Revilla-León et al. [72]	Restorative dentistry	2022
Li et al. [73]	Restorative dentistry	2022
Aminoshariae et al. [74]	Endodontics	2021
Karobari et al. [37]	Endodontics	2023
Kierce et al. [75]	Periodontal disease	2023
Mohammad-Rahimi et al. [76]	Periodontal disease	2022
Singi et al. [77]	Prosthetic dentistry	2022
Revilla-León et al. [78]	Implantology	2023
Monill-González et al. [79]	Orthodontics	2021
Vishwanathaiah et al. [80]	Pedodontics	2023

## Data Availability

All data are available from the corresponding author upon reasonable request.

## Abbreviations

AI	Artificial intelligence
NNs	Neural networks
ML	Machine learning
CNNs	Convolutional neural networks
ANNs	Artificial neural networks
IOU	Intersection-over-union
NILT	Near-infrared light transillumination
CBCT	Cone Beam Computed Tomography
ALs	Apical lesions
PPV	Positive predictive value
NPV	Negative predictive value
VRF	Vertical root fractures
WL	Working length
RBL	Radiographic bone loss
